# Janus kinase-signal transducer and activator of transcription signaling pathway in the ocular cells of rat fetuses exposed to maternal saturated fat ingestion

**DOI:** 10.1590/1806-9282.20231167

**Published:** 2024-04-22

**Authors:** Lorrany da Silva Avanci, Thiago Guedes Pinto, Daniel Vitor de Souza, Lais Vales Mennitti, Daniel Araki Ribeiro, Luciana Pisani

**Affiliations:** 1 Universidade Federal de São Paulo, Department of Biosciences – Santos (SP), Brazil.

**Keywords:** JAK/STAT pathway, Eye, Fatty acids

## Abstract

**OBJECTIVE:**

The aim of this study was to analyze possible alterations (morphological and inflammatory) in the ocular cells of fetuses from mothers with insulin resistance exposed to saturated fatty acids through the period of pregnancy.

**METHODS:**

Wistar female rats were induced to develop insulin resistance before pregnancy. Fetuses' skulls were collected on the 20th day of intrauterine life. The rats were separated on the first day of management into two groups according to the diet applied: control group (C): diet containing soybean oil as a source of fat; and saturated fatty acid group (S): diet containing butter as a source of fat.

**RESULTS:**

Histological and immunohistochemical analyses have been conducted. The immunohistochemical analyses of interleukin 6, suppressor of cytokine signaling, 3 and signal transducer and activator of transcription 3 did not demonstrate alterations in the expression of proteins in the fetuses of mothers fed with a saturated fatty diet. Moreover, no histopathological changes were noticed between groups.

**CONCLUSION:**

The saturated fatty diet does not induce tissue changes or activate the Janus kinase/signal transducer and activator of transcription signaling pathway during eye development in the fetuses of mothers with insulin resistance.

## INTRODUCTION

Several studies have indicated that dietary behavior and health conditions impact not only the health of mothers but also fetus, therefore affecting its development^1^. Even though fetal development is basically driven by the program encoded in its genome, the epigenetic regulation of its growth is directly influenced by the intrauterine environment^2^. In this context, the fetus' health depends on the supply of nutrients and oxygen from the mother, and a malfunction of this system in the gestational period may lead to metabolic effects throughout the fetus' life, encompassing childhood and adolescence^3^.

According to some authors, a significant imbalance in macronutrient intake may lead to health harm. Saturated fatty acids (such as lauric acid, myristic acid, palmitic acid, and stearic acid), for instance, may induce transcription of factor κB (nuclear factor-kappa B) and culminate in the activation of proinflammatory genes (such as cyclooxygenase-2)^4^. In this regard, this cascade of events could represent an additional factor in increasing cholesterol levels and triggering cardiovascular diseases^5^.

Although in past decades dietary guidance has almost universally advocated reducing the intake of total and saturated fat, these recommendations, as well as the link between fat consumption and the risk of human disease, have been among the most vexed questions in public health in order to fully understand whether dietary fats are harmful^6^.

The Janus kinase-signal transducer and activator of transcription (JAK-STAT) signaling pathway (one of the main signaling pathways) in eukaryotic cells might be useful as it is activated in several growth and developmental processes in multiple tissues in order to control cell proliferation, differentiation, survival, and apoptosis^7^. It is activated when certain cytokines bind to the receptor complex, JAK1 and JAK3, allowing the recruitment and phosphorylation of STAT3, which dimerizes and enters the nucleus, activating a transcription program of the target genes, and stabilizing the receptor complex^8^. Also, such binding may activate the signaling pathway of mitogen-activated protein kinase and phosphoinositide 3-kinase signaling, which may induce the transcription of certain cytokine signaling suppressors (SOCS), therefore inhibiting the JAK/STAT pathway^9^. Besides, the proinflammatory cytokine interleukin 6 (IL-6) demonstrates JAK-STAT signaling dependence, which justifies our choice to study such biomarkers in the current study^10^.

Of particular importance, *Drosophila melanogaster*'s eyes have been extensively used as a platform to understand signaling pathways, and several reports have demonstrated that the JAK/STAT signaling pathway plays pleiotropic roles in *Drosophila* eye development. The biological mechanism involves activation (dependent on the ligand Upd) of the mentioned pathway in eye tissues during development^8^. On the contrary, retinal degenerative diseases are a major cause of severe visual impairment or blindness in humans, and genes and proteins of the JAK/STAT signaling pathway have been shown to play an important role in models of retinal degeneration, which justifies the conduct of studies within the field^9^.

The purpose of this study was to evaluate the histopathological changes as well as the expression of the JAK/STAT pathway in the eyes of rat fetuses from dams with insulin resistance treated with a saturated-fat normolipidic diet during pregnancy.

## METHODS

### Animals and experimental design

Every procedure of the current study was carried out following the International Standards for Research involving Animals. Regarding animals' features, a total of 13 2-month-old virgin Wistar female rats were obtained from Centro de Desenvolvimento de Modelos Experimentais para Medicina e Biologia (CEDEME). Concerning environmental conditions, the rats were kept under light-cycle conditions (12 h light and 12 h dark) at 24°C±1°C with food and water *ad libitum*. This study was approved by the Animal Ethics Committee under Protocol # 8298100720. Furthermore, female rats were induced to develop insulin resistance through high-fat (HF) diet preconception and mated when they were 4 months old, and on the following day, the presence of spermatozoa in the vaginal lumen was verified with the aid of a microscope (through the introduction of a small amount of 0.9% saline solution into the vagina with posterior aspiration with a dropper). After likely conception confirmation, the females were kept in individual plastic cages and distributed sequentially into two different groups. During pregnancy, each group received a different experimental diet as follows: control group (C): diet containing soybean oil as a source of fat (n=5) and saturated fatty diet group (S): diet containing butter as a source of fat (n=5). The recommendations of the American Institute of Nutrition (AIN-93G) were followed in the composition of the diets^11^. The experimental design was established in a previous study^12^. Also, body weight and food intake were recorded weekly, considering both animal groups.

On the 20th day of gestation (from a total of 21–22 days), the pregnant rats were euthanized to obtain the fetuses. The rats were anesthetized with isoflurane (Isoflurane^®^; BioChimico Ltda, Itatiaia, RJ, Brazil) by inhalation and then decapitated after 4 h of fasting. Fetuses were collected (regardless of sex) by caesarean section and euthanized by decapitation. Fetal skulls were collected for histological analysis of ocular tissue.

### Histopathological analysis

After collecting the skulls, the tissue was embedded in paraffin, and then histological sections of 3 μm thickness were performed. The following parameters were evaluated: cell morphology, evaluation of cell nuclei, presence of inflammatory infiltrate, presence of hemorrhage, and atypical cells.

### Immunohistochemical analysis

Histological sections were serially cut in the microtome sections with a 3-μm thickness, and then the slides were deparaffinized in xylene, rehydrated in ethanol (99.5%), and pretreated with citric acid buffer (10 nM, pH 6, 0.1 M citric acid, Synth^®^, São Paulo, Brazil; 0.1 M sodium citrate—Synth^®^, São Paulo, Brazil), and antigenic retrieval was performed in microwaves (for 3 cycles of 5 min). Primary antibodies (Santa Cruz Biotechnology, Inc., USA) were used as follows: IL-6 dilution 1:150; STAT 3 diluted 1:100; and SOCS 3 diluted 1:200, applied to slides and incubated overnight at 4°C. Following day, the slides were washed twice with phosphate-buffered saline (PBS) and incubated with biotinylated secondary antibody (Starr Trek Universal HRP Detection Kit, Biocare Medical^®^, USA) for 30 min, washed with PBS and incubated with streptoavidin conjugated with hydrogen peroxide for 30 min, and then stained with 3,3-diaminobenzidine, (DAB, 0.05%—DAKO North America Inc.^®^, California, USA). Finally, counterstaining with hematoxylin was performed, and the slides were mounted with resin (Entellan^®^ new, Merck, Germany).

## RESULTS

### Maternal oral glucose tolerance test and area under the curve

**Figure 1:**
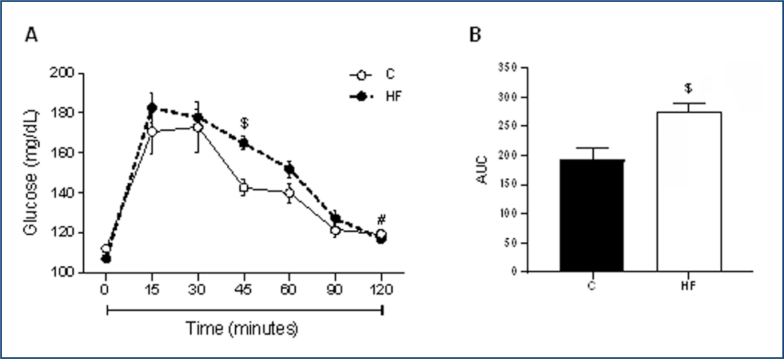
Oral glucose tolerance test and area under the curve in the female rats after 8 weeks of dietary treatment. **(A)** Glycemia at baseline, 15, 30, 45, 60, 90, and 120 min after 2 g of glucose/kg of body weight, and **(B)** area under the curve in the female rats. C: female rats fed a control diet; and HF: female rats fed a high-fat diet. $p≤0.05 versus C and zero (controls) and #p≤0.05 HF basal versus HF after 120 min.

Figure 1 illustrates the increased area under the curve (AUC) (Figure 1B) and blood glucose levels after 45 min of oral glucose administration (Figure 1A) in dams that received the HF diet (43% lipids) during the pregestational period. In contrast to the dams in the control group, baseline glucose levels (time 0) were not recovered in the HF group after 120 min from the start of the test. These findings have shown that consuming the HF diet for 8 weeks before pregnancy impaired the glycemic response in the female rats, reducing oral glucose tolerance and influencing the development of insulin resistance.

### Histopathological analysis

**Figure 2:**
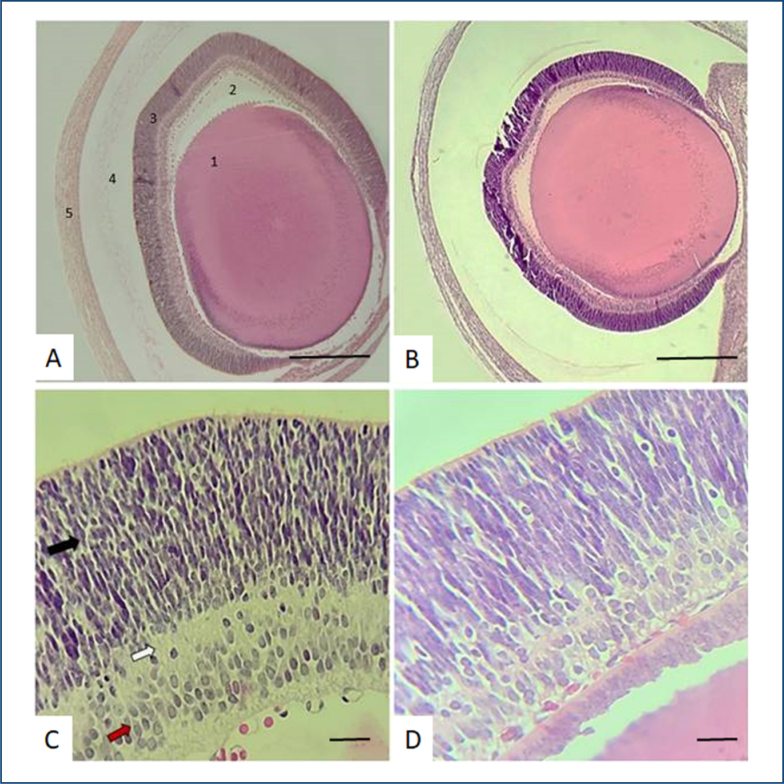
Ocular tissue in the control group **(A):** 1 crystalline; 2 vitreous humor; 3 retina; 4 posterior chamber; 5 cornea; and treated group **(B)**: no remarkable changes. **(C)** High magnification of the control group and the treated group **(D):** the black arrow points to the photoreceptor cone cell layer; the white arrow points to the interneuron layer; and the red arrow points to the location of the retinal ganglion cells. No change was observed in cell morphology between the two analyzed groups. Hematoxylin and eosin analysis. Scale bar=34 μm.

The analysis of the ocular tissue of Wistar rat fetuses, as shown in Figure 2, occurred through observation of the cornea, retina, vitreous humor, and crystalline lens in both the control group (A) and the group treated with saturated fatty acid (B). The groups were mutually compared, and it was not possible to observe changes in all analyzed tissues in this setting. In the lens, the integrity of the fibers arranged in layers, capsules, and epithelium was observed. Also, the vitreous humor (composed of an aqueous substance containing collagen fibers, hyaluronic acid, and the hyaloid vascular system) presented preserved structure with no changes after the treatment period. Retinal ganglion cells also showed preserved cellular morphology and structure after the experimental treatment (Figures 2C and 2D).

### Immunohistochemical analysis

**Figure 3:**
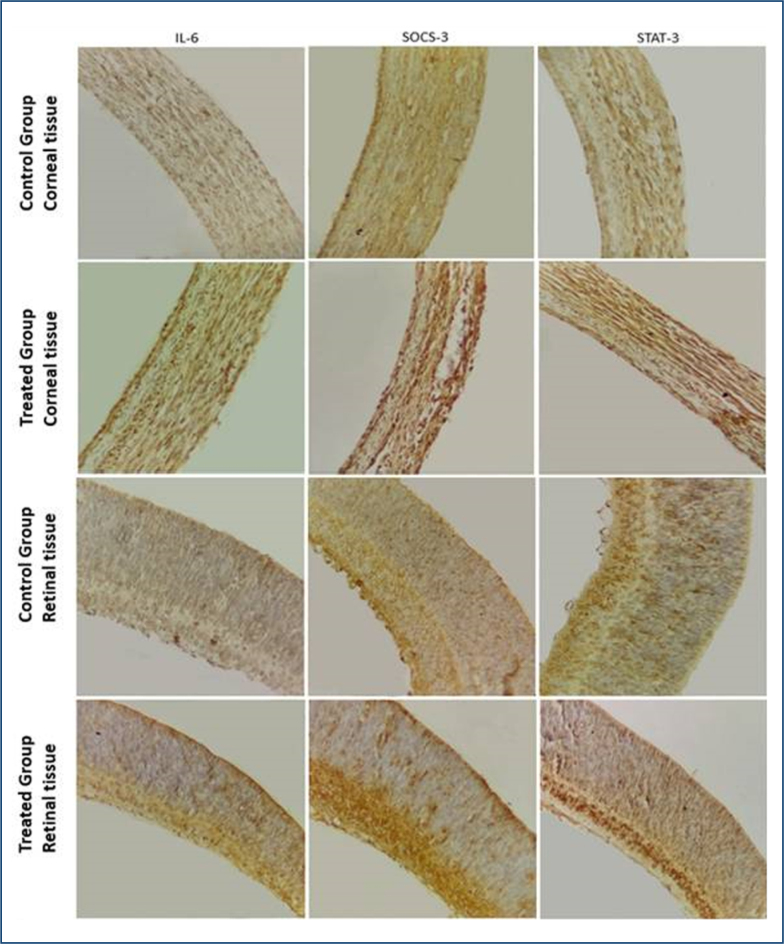
Corneal and retinal tissue immunohistochemistry for interleukin, suppressor of cytokine signaling, and signal transducer and activator of transcription 3. Immunohistochemical stain.

Immunohistochemical analyses were performed for the detection of IL-6, STAT-3, and SOCS-3. In Figure 3, the experimental groups and the control group did not show significant relevance between them, since the expressions of pro-inflammatory cytokine IL-6, STAT-3 protein, and SOCS-3 protein were observed in the retinal and corneal tissues of both groups. In the other tissues studied in this research, expression was not observed for the tests performed.

## DISCUSSION

Some review papers have suggested that saturated fatty diets have no effect on cardiovascular disease, cardiovascular mortality, or even total mortality^5^. Despite the fact that medical literature is still full of articles arguing opposing positions, the recommendation to limit dietary saturated fatty acid intake has persisted, even though most recent meta-analyses of randomized trials and observational studies found no beneficial effects of reducing saturated fatty diet intake on human diseases. Some studies even suggest that saturated fatty acid intake may lead to protective effects against stroke^6^.

It is known that the development of ocular tissue in rats is similar to the development of ocular tissue in humans and, in both species, development, biochemical, and metabolic processes are widely involved^13^. Regarding the human eye structure, it is important to highlight that it is produced from the coordinated development of multiple tissues (neuroectodermal, ectodermal, and mesodermal). Also, three layers can be distinguished in this complex organ: the outer region, composed of the cornea and the sclera; the middle one, composed of the iris, ciliary body, and choroid; and finally, the inner layer, which is composed of the retina with two different cell types (rods and cones).

According to a great number of studies, harmful effects of maternal insulin resistance and consumption of high amount of saturated fatty acids, especially during pregnancy or lactation, have been observed^14,15^. Therefore, an increased consumption of saturated fatty acids may lead to increased concentrations of glucose, insulin, leptin, tumor necrosis factor-α (TNF-α), monocyte chemoattractant protein-1, IL-6, NF-κB, and Toll-like receptor 4 activation^16,17^. In this sense, it has also been demonstrated that the increased production of TNF-α in the vascular endothelium is probably related to the risk of cardiovascular disease development, which involves inflammatory processes that may ultimately affect the skeletal muscle and the liver negatively^18^. Furthermore, high concentrations of TNF-α demonstrated a positive association with newborns from mothers with pre-eclampsia^19^.

Currently, less information is available on whether and to what exent a saturated fatty diet is able to induce changes following ocular development. In our study, no histopathological changes were observed in all tissues analyzed in the experimental and control groups in this setting. In addition, no remarkable changes were noticed in the IL-6, STAT-3, and SOCS-3 expressions. By comparison, some authors have postulated that a high content of saturated fat, cholesterol, and sugar significantly increased retinal leukocyte accumulation and endothelial injury in streptozotocin-diabetic rats^20^. The consumption of diets containing higher palmitic acid concentrations modulates rats' mucus granule surfaces in their goblet cells^21^. Taken as a whole, we assume that the maternal diet during gestation with a normolipidic diet based on saturated fatty acids did not appear to trigger significant changes in the ocular tissue of fetuses (such as other cell morphology changes, cell atypicity, or the presence of degenerations), even in the presence of maternal insulin resistance.

## CONCLUSION

The saturated fatty diet does not induce tissue changes that activate the JAK/STAT signaling pathway during eye development in the fetuses of mothers fed a saturated-fat normolipidic diet. Given the absence of similar studies for possible comparisons, it can be concluded that, despite the previously related high inflammatory potential of fatty acids and their direct exposure to fetus health, the normolipid maternal diet, regardless of the type of fatty acid intake, does not induce eye damage in the fetuses, emphasizing the importance of diet quality during pregnancy. Further studies involving other cellular signaling pathways within the field are necessary to elucidate the issue.
